# Pulsed field ablation of atrial fibrillation via jugular transseptal puncture in a patient with dextrocardia and interrupted inferior vena cava: a case report

**DOI:** 10.1093/ehjcr/ytaf577

**Published:** 2025-10-31

**Authors:** Marco Scaglione, Enrico Guido Spinoni, Francesco Geuna, Andrea Lamanna, Alberto Battaglia

**Affiliations:** Electrophysiology Laboratory, SC Cardiologia, Cardinal Massaia Hospital, Corso Dante Alighieri 202, 14100 Asti, Italy; Electrophysiology Laboratory, SC Cardiologia, Cardinal Massaia Hospital, Corso Dante Alighieri 202, 14100 Asti, Italy; Electrophysiology Laboratory, SC Cardiologia, Cardinal Massaia Hospital, Corso Dante Alighieri 202, 14100 Asti, Italy; Johnson & Johnson MEDICAL S.p.A., Via del Mare 56, 0071 Pomezia, Italy; Electrophysiology Laboratory, SC Cardiologia, Cardinal Massaia Hospital, Corso Dante Alighieri 202, 14100 Asti, Italy

**Keywords:** Atrial fibrillation, PFA, Dextrocardia, Interrupted inferior vena cava, Transjugular transseptal puncture

## Abstract

**Background:**

Situs viscerum inversus with dextrocardia and interrupted inferior vena cava (I-IVC) are rare congenital diseases that might challenge interventional procedures, such as transcatheter ablation for atrial fibrillation (AF), when transseptal puncture (TSP) is required for left atrial access. Recently, one-shot pulsed field ablation (PFA) has emerged as a safe and effective energy modality for AF ablation. We present a case report on jugular TSP guiding one-shot PFA in a patient presenting dextrocardia and an I-IVC, addressing the unique anatomical challenges.

**Case summary:**

A 52-year-old male with situs viscerum inversus with dextrocardia and I-IVC presented to our institution with symptomatic paroxysmal AF. Pre-procedural evaluation included transthoracic echocardiography and cardiac magnetic resonance (CMR) imaging. The PFA procedure was successfully performed under general anaesthesia guided by transoesophageal echocardiography (TOE), fluoroscopy, electroanatomical mapping and merging with CMR imaging. A transjugular TSP was specifically employed for vascular access due to the complex anatomy, allowing for efficient left atrial access and delivery of the ablation therapy.

**Discussion:**

Our case report highlights the role of multimodality pre-procedural imaging and the necessity of adapting procedural techniques, such as jugular TSP, to safely and effectively perform AF ablation in patients with complex congenital anatomy, as dextrocardia with I-IVC. The use of one-shot variable loop PFA catheter in such a challenging anatomical scenario allows an effective and safe treatment for transcatheter AF ablation in this case.

Learning points

- In patients with complex anatomy, such as dextrocardia with interrupted inferior vena cava, imaging integration plays a key role in the procedural planning of atrial fibrillation ablation.
- Pulsed field ablation with a one-shot variable loop catheter represents a valuable strategy for atrial fibrillation ablation in patients with complex anatomy and requiring jugular transseptal puncture

## Introduction

Situs viscerum inversus with dextrocardia and interrupted inferior vena cava (I-IVC) are exceptionally rare congenital malformations.^1,2^ The coexistence of these conditions presents a significant procedural challenge in transcatheter ablation of atrial fibrillation (AF), particularly when transseptal puncture (TSP) is required to access the functional left atrium (LA).

Recently, one-shot pulsed field ablation (PFA) has emerged as a safe and effective energy modality for AF ablation.^3,4^

In this report, we describe a transjugular approach for guiding one-shot PFA in a patient with both dextrocardia and an I-IVC.

## Summary figure

**Figure:**
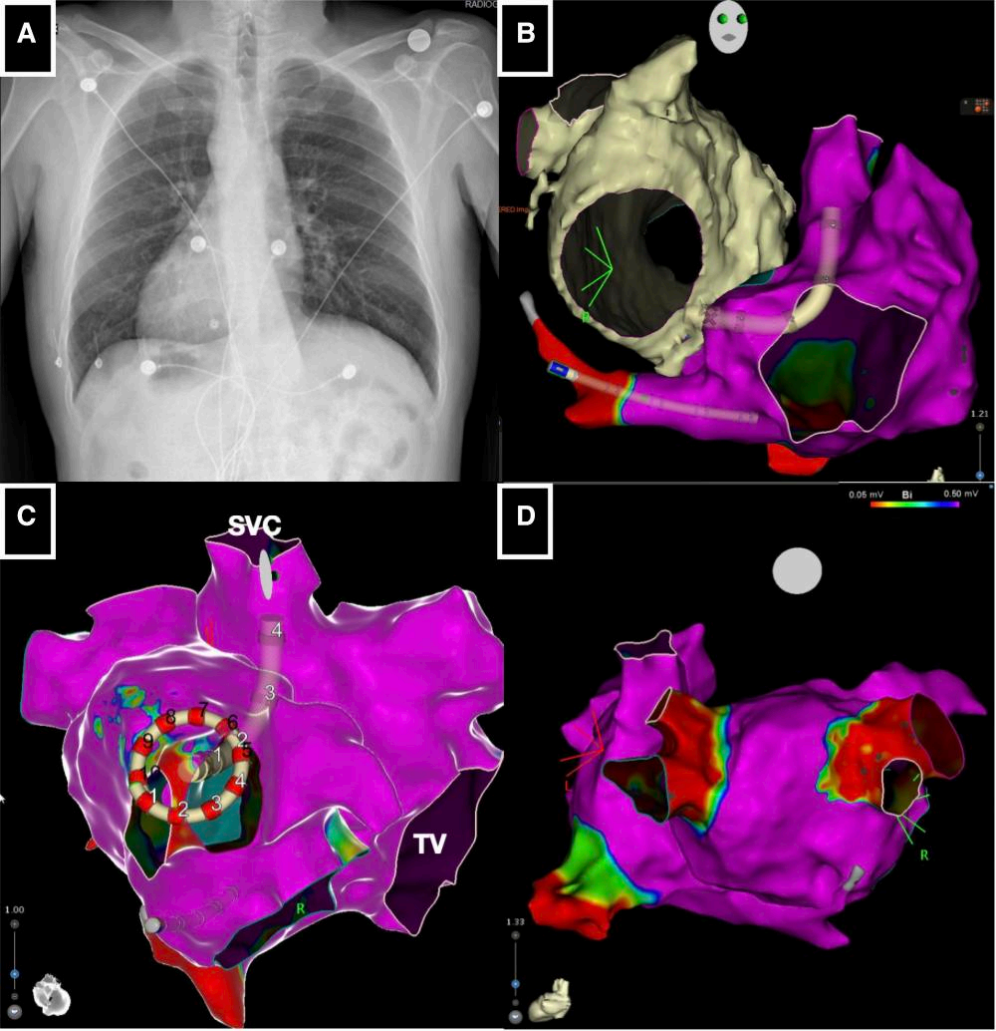
Pulsed field ablation of atrial fibrillation in a patient with situs viscerum inversus, dextrocardia, and interrupted inferior vena. (*A*) Standard chest radiography highlighting dextrocardia. (*B*) Electroanatomical mapping (RAO projection mimicking LAO) with cardiac magnetic resonance integration guiding the positioning of the VIZIGO sheath at the fossa ovalis. (*C*) Electroanatomical mapping guiding Varipulse catheter positioning within anatomical right pulmonary veins. (*D*) Electroanatomical mapping post-ablation confirming complete pulmonary vein isolation with PFA.

## Case presentation

**Figure 1:**
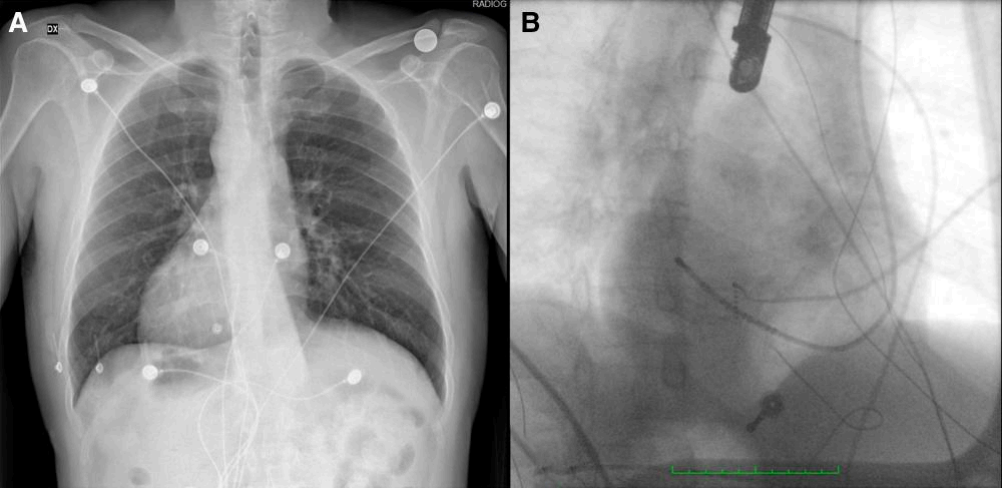
(*A*) Standard chest radiography highlighting dextrocardia. (*B*) Fluoroscopy right anterior oblique (RAO) projection with decapolar catheter inserted in the coronary sinus mimicking left anterior oblique (LAO) projection.

A 52-year-old man presented to our institution with episodes of symptomatic paroxysmal AF. He had a pre-existing diagnosis of situs viscerum inversus with dextrocardia (*Figure 1A*). Transthoracic echocardiography during his initial cardiological evaluation showed a preserved ejection fraction (EF; 65%), mild mitral regurgitation, and confirmed dextrocardia. Anti-arrhythmic drug therapy with flecainide 100 mg twice daily (b.i.d.) was initiated. Due to symptomatic relapse of arrhythmia, an AF ablation procedure was planned. Upon in-hospital admission, the patient was in stable sinus rhythm (SR).

The pre-procedural workflow included cardiac magnetic resonance (CMR) imaging for the reconstruction of the heart chambers and major vessels. The CMR scan revealed the absence of IVC connection to the functional right atrium, consistent with suspected I-IVC and the presence of azygos vein continuation. Ablation was performed the following day under general anaesthesia, guided by transoesophageal echocardiography (TOE), fluoroscopy, electroanatomical (EAM) mapping, and merging of CMR imaging.

**Figure 2:**
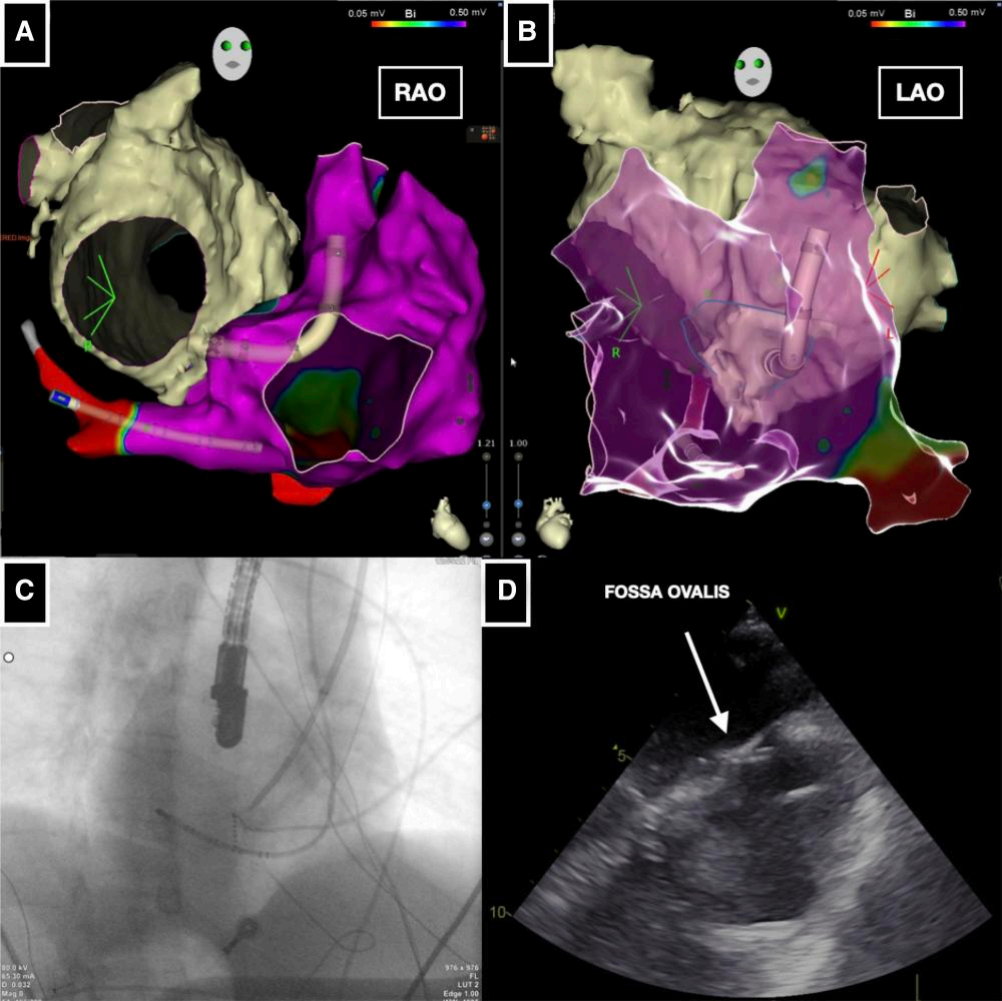
(*A* and *B*) Electroanatomical mapping [RAO projection mimicking LAO projection (*A*) and LAO projection mimicking RAO projection (*B*)] with cardiac magnetic resonance integration guiding the positioning of the VIZIGO sheath at the fossa ovalis. (*C* and *D*) Fluoroscopy RAO projection (*C*) and transoesophageal echocardiography (*D*) guiding the positioning of Fast-Cath long sheath at the fossa ovalis.

Right femoral vein access was obtained under echocardiographic guidance. Contrast medium was then injected through the sheath, confirming the suspected I-IVC and the presence of azygos vein continuation. Subsequently, ultrasound-guided accesses were obtained via the left internal jugular vein and the left subclavian vein. A decapolar deflectable catheter was advanced into the coronary sinus via the left subclavian vein access (*Figure 1B*). At this time, a bi-directional guiding sheath (CARTO VIZIGO™, Biosense Webster, Irvine, CA, USA) with integrated visualization on the EAM mapping system (CARTO3, Biosense Webster) was introduced via the jugular vein access. Reconstruction of the functional right atrium was then performed using a multipolar mapping catheter (Pentaray, Biosense Webster). The obtained EAM was subsequently merged with the previously acquired CMR imaging (*Figure 2A* and *B*) to guide and identify the optimal site for TSP.

A manually curved Brockenbrough needle (BRK, St. Jude Medical) was advanced into the right atrium via the VIZIGO™ sheath, which was inserted through the right jugular vein, under fluoroscopic, EAM, and TOE guidance. However, it was not possible to adequately deflect the VIZIGO™ sheath to engage the fossa ovalis for the TSP. Consequently, it was replaced with an 8.5 Fr transseptal long sheath (Fast-Cath 60 cm, Mullins Curve, St. Jude Medical, Minneapolis, MN, USA). The Brockenbrough needle (BRK, St. Jude Medical) was then advanced via the left jugular vein into the right atrium through this new sheath (*Figure 2C*). The key point was to manually bend the needle to create a very large curve being able to engage and support the TSP puncture, as confirmed by TOE (*Figure 2D*).

**Figure 3:**
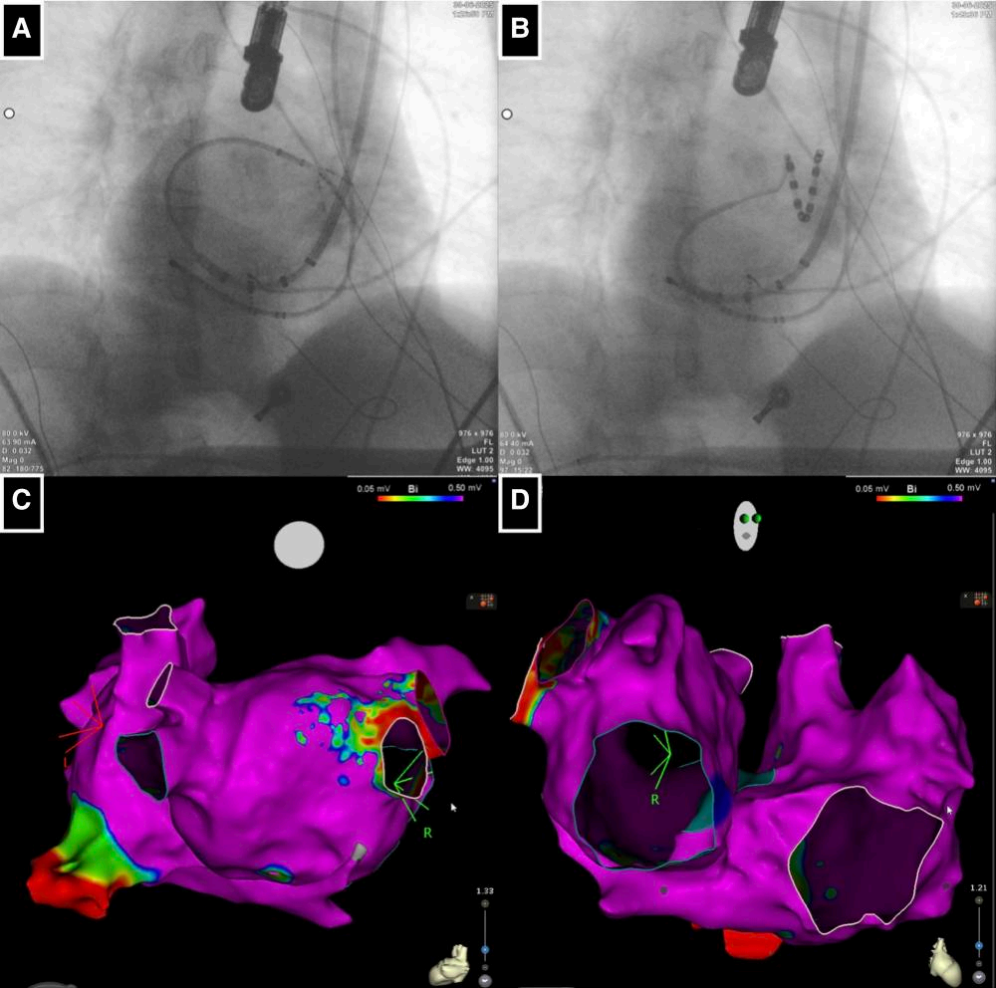
(*A*) Fluoroscopy RAO projection guiding mapping catheter manoeuvres in the left atrium. (*B*) Fluoroscopy RAO projection guiding variable loop pulsed field ablation catheter manoeuvres in the left atrium. (*C* and *D*) Electroanatomical reconstruction of the left and right atrium in postero-anterior (PA) and RAO projection.

**Figure 4:**
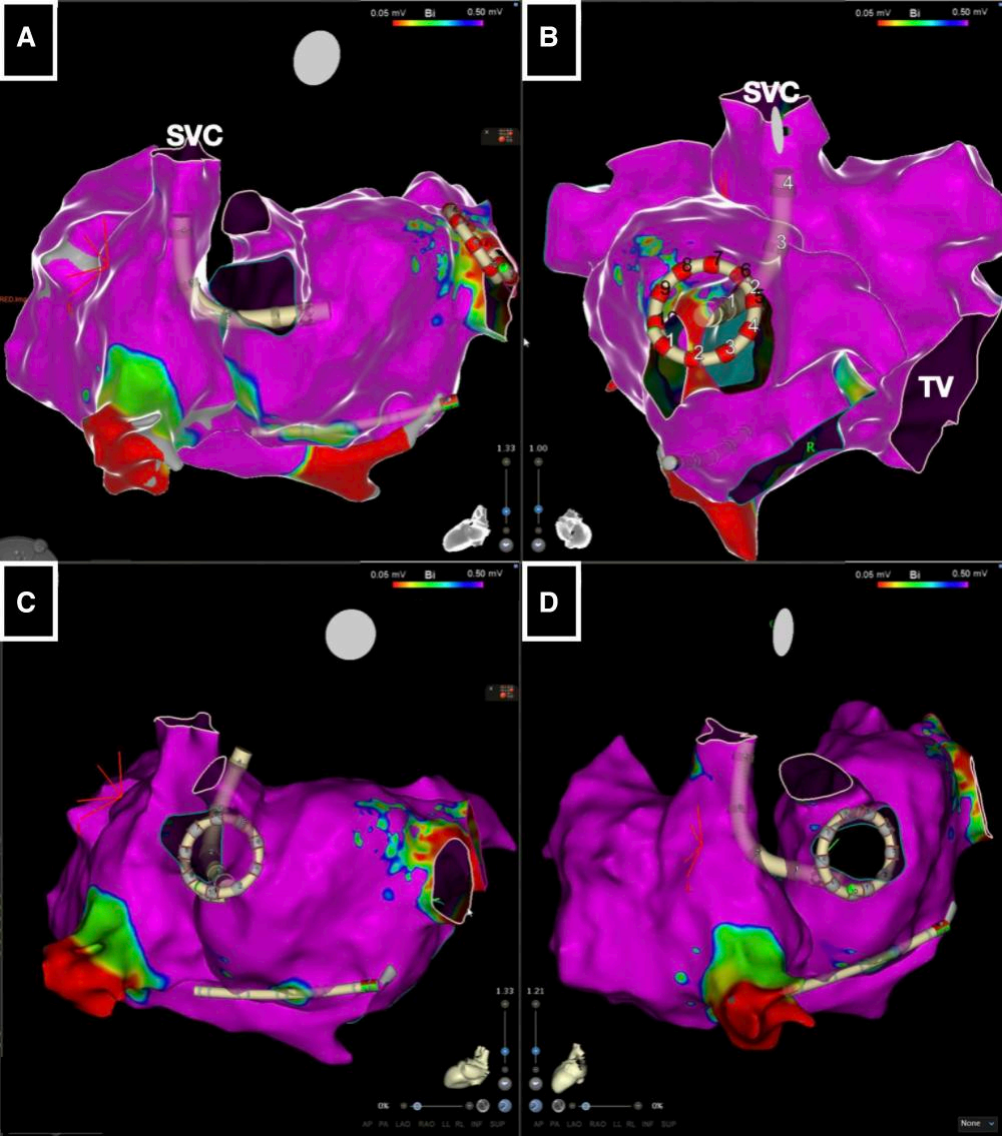
(*A–D*) Electroanatomical map projections guiding variable loop pulsed field ablation catheter positioning in the pulmonary veins for transcatheter ablation. SVC, superior vena cava; TV, tricuspid valve.

Once left atrial (LA) access was achieved, the long sheath was replaced with the VIZIGO™ sheath to allow its deflection into the LA to manoeuvre better both the mapping and the ablation catheter (*Figure 3A* and *B*). Once the EAM of the LA was reconstructed with the mapping catheter, it was merged with the CMR imaging rendering by CARTO-Merge software (Biosense Webster) (*Figure 3C* and *D*). Considering that a point-by-point ablation strategy might be challenging due to the complexity of LA access and anatomical relationships, a single-shot variable-loop PFA catheter was selected (Varipulse™, Biosense Webster), delivering four PFA pulses per vein, as per the current protocol^5^ (*Figure 4*).

**Figure 5:**
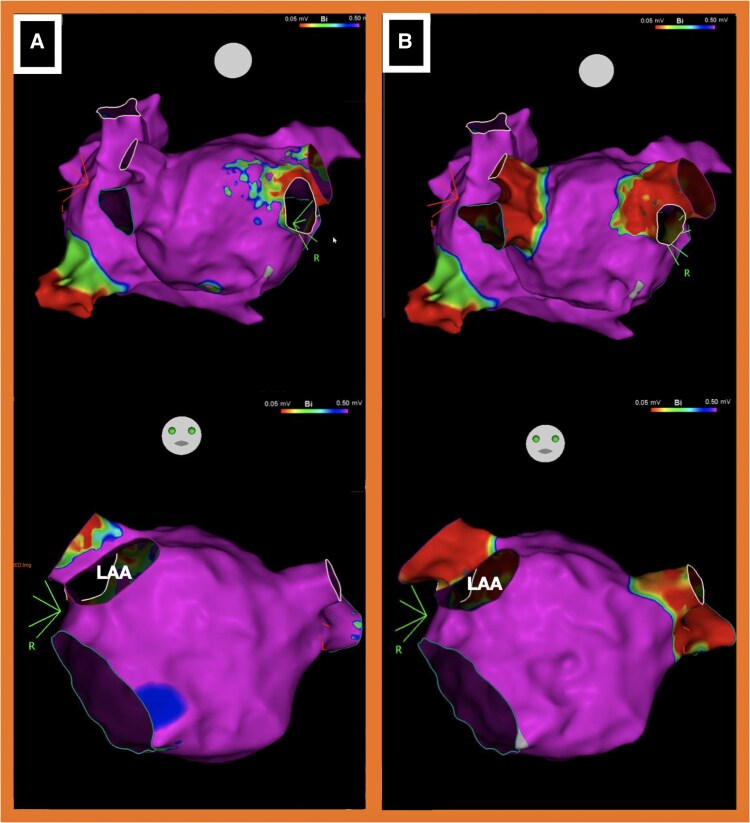
(*A*) Electroanatomical map before atrial fibrillation ablation (upper figure PA projection, lower figure AP projection). (*B*) Electroanatomical map after atrial fibrillation ablation (upper figure PA projection, lower figure AP projection) confirming pulmonary vein isolation. LAA, left atrial appendage.

Following the ablation phase, a new EAM was performed, confirming complete isolation of all four pulmonary veins (*Figure 5B*). The skin-to-skin procedure time was 150 min. Post-procedure, transthoracic echocardiography was conducted, which excluded the presence of a significant pericardial effusion. The patient was monitored for 24 h post-procedure. Thoracic radiography performed the following day revealed no evidence of pneumothorax. The patient was discharged home the day after the ablation procedure.

## Discussion

In patients with IVC interruption, which precludes the use of standard femoral vein access for electrophysiology procedures, several strategies have been described, including the superior approach (jugular or subclavian vein), transhepatic access, and transaortic access.^6–10^ We opted for the superior transjugular approach due to its favourable risk–benefit profile, supported by a low reported rate of major complications and our centre’s extensive expertise in echo-guided jugular vein puncture

Use of right internal jugular vein for TSP was first described by Lim *et al*. for AF ablation in three patients with I-IVC, and the results were subsequently reproduced by small case series from different groups employing various approaches.^6–10^ In most of these cases, fluoroscopy and intracardiac echocardiography (ICE) guidance were used to detect the right puncture site. Transoesophageal echocardiography is reported as a safe alternative. In these cases, the TSP has been performed with a Brockenbrough needle manually curved or using a radiofrequency (RF) needle or RF wire.^6–10^

A single case report by Xu *et al*.^11^ described TSP by ICE guidance in a patient with I-IVC and dextrocardia.

In our case report, the patient exhibited a highly complex anatomy, characterized by an I-IVC and situs viscerum inversus with dextrocardia. Our procedural workflow involved the integration of multiple imaging modalities (CMR, TOE, and fluoroscopy combined with EAM). By combining these techniques, we were able to effectively guide the procedure, identify the optimal site, and perform the TSP safely. Our workflow for jugular TSP included:

- Ultrasound-guided left jugular and subclavian vein access
- Decapolar catheter advancement by the left subclavian vein in coronary sinus with an initial EAM reconstruction of the right atrium
- Positioning of mapping catheter through the jugular vein and complete reconstruction of the right atrium allowing merging and alignment with corresponding CMR rendering
- Positioning of VIZIGO™ sheath visualized by EAM in the right atrium on the fossa ovalis, confirmed by fluoroscopy and TOE imaging
- Substitution of VIZIGO™ sheath with Fast-Cath long sheath, which provided substantial support for needle advancement for TSP

Numerous studies have recently been published on the safety and efficacy of variable-loop one-shot PFA catheters.^12–14^ Given these results, and considering the patient’s exceptionally complex anatomy, which could potentially influence the manoeuvrability of the ablation catheter and impact ablation outcomes in terms of safety and efficacy, ablation was performed using a single-shot variable-loop PFA catheter. Furthermore, the curvature of the catheter, supported by the bi-directional long sheath, enabled the effective ablation even in LA regions that would have otherwise been extremely challenging to target, such as the anatomically ‘left pulmonary veins’ (*Figure 3B*). The critical steps of this procedure were the following: (i) the integration of imaging (fluoroscopy, CMR, EAM, and TOE) which allowed effective and safe access to the target chamber; and (ii) the availability of the one-shot ablation tool, which offered good manoeuvrability and a favourable safe profile, enabling a procedure in such complex anatomy.

To the best of our knowledge, there have been no previously published cases of AF ablation using PFA performed via jugular TSP in patients with dextrocardia and an I-IVC.

## Conclusions

In patients with complex anatomy, as dextrocardia with I-IVC, imaging integrations and the use of variable loop single-shot PFA catheter were effective in carrying out AF ablation by jugular TSP.

## Lead author biography


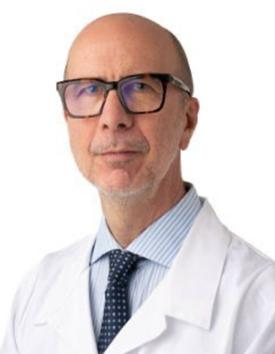


Professor Marco Scaglione is an interventional arrhythmologist and director of the electrophysiology laboratory in Asti. The centre he leads is a national reference for interventional electrophysiology procedures in adult patients, paediatric populations, and adults with congenital heart disease.

## Data Availability

The data underlying this article will be shared on reasonable request to the authors.

## Notes

**Consent:** The authors state that written consent for publication of this case report, including images and associated text, has been obtained from the patient in line with the COPE guidelines.
